# 4462 Effects of Injectable, Erythropoietin and Glucocorticoids Combinational Therapy on Erythrocyte Sedimentation Rate Following Spinal Cord Injury

**DOI:** 10.1017/cts.2020.176

**Published:** 2020-07-29

**Authors:** Anna Nia, Kamil Khanipov, George Golovko

**Affiliations:** 1 University of Texas Medical Branch

## Abstract

OBJECTIVES/GOALS: Inflammation following traumatic injury to the spinal cord persists long after the primary insult and is known to increase complication rates and prolong recovery time. We investigated the effects of Erythropoietin (EPO) in combination with Glucocorticoids on the levels of erythrocyte sedimentation rate (ESR), an overall measure of inflammation. METHODS/STUDY POPULATION: Electronic medical records from approximately 38 million patients in 27 Healthcare Organizations were analyzed using the TriNetX Analytics platform. Patients with spinal cord injuries (SCI) were defined with the ICD-10 code, G95 and two unique cohorts were defined for patients treated with injectable EPO in combination with injectable Glucocorticoids within 6 months of SCI or only injectable Glucocorticoids with no injectable EPO. ESR rates were queried from patient cohorts to evaluate the potential effects of the two treatment pathways on the ESR. Most recent lab results within 6 months before initiating treatment and 1-year post-treatment were defined as “before” and “after” treatment, respectively. Changes in ESR lab results were evaluated using unpaired t-test with Welch’s Correction. RESULTS/ANTICIPATED RESULTS: A total of 14,370 patients satisfied the inclusion criteria. 89 patients were treated with injectable EPO in combination with Glucocorticoids within 6 months of SCI. The ESR lab results were available for 33 patients before treatment with a mean of 63±33 mm/h. The ESR lab results were available for 22 patients after treatment with a mean of 51.7±34.1 mm/h. 14,281 patients were treated with Glucocorticoids (no injectable EPO) within 6 months of SCI. The ESR lab results were available for 2,042 patients before treatment with a mean of 29.2±30.5 mm/h. The ESR lab results were available for 2,184 patients after treatment with a mean of 32.6±30 mm/h. Patients treated with combinational therapy showed a reduction in ESR of 11.3 mm/h, while those treated with only Glucocorticoids showed an increase in ESR of 3.4 mm/h. DISCUSSION/SIGNIFICANCE OF IMPACT: The present results demonstrated that combinational therapy with injectable, EPO and glucocorticoids exhibited a significant reduction in ESR level. The study suggests that EPO and glucocorticoid might have a synergistic effect on reducing the inflammation following SCI. This approach might help reduce the therapeutic dose of glucocorticoids. Conflict of Interest Description: The authors declare that they have no competing interests. CONFLICT OF INTEREST DESCRIPTION: The authors declare that they have no competing interests.

